# Identifying links between monsoon variability and rice production in India through machine learning

**DOI:** 10.1038/s41598-023-27752-8

**Published:** 2023-02-10

**Authors:** Christopher Bowden, Timothy Foster, Ben Parkes

**Affiliations:** Department of Mechanical, Aerospace and Civil Engineering, University of Manchester, Manchester, M13 9PL UK

**Keywords:** Climate sciences, Environmental sciences

## Abstract

Climate change poses a major threat to global food security. Agricultural systems that rely on monsoon rainfall are especially vulnerable to changes in climate variability. This paper uses machine learning to deepen understanding of how monsoon variability impacts agricultural productivity. We demonstrate that random forest modelling is effective in representing rice production variability in response to monsoon weather variability. Our random forest modelling found monsoon weather predictors explain similar levels of detrended anomaly variation in both rice yield (33%) and area harvested (35%). The role of weather in explaining harvested rice area highlights that production area changes are an important pathway through which weather extremes impact agricultural productivity, which may exacerbate losses that occur through changes in per-area yields. We find that downwelling shortwave radiation flux is the most important weather variable in explaining variation in yield anomalies, with proportion of area under irrigation being the most important predictor overall. Machine learning modelling is capable of representing crop-climate variability in monsoonal agriculture and reveals additional information compared to traditional parametric models. For example, non-linear yield and area responses of irrigation, monsoon onset and season length all match biophysical expectations. Overall, we find that random forest modelling can reveal complex non-linearities and interactions between climate and rice production variability.

## Introduction

Agriculture is critically important to food security in South Asia, a region that is home to nearly half the malnourished population of the world^1^. Overall, 56% of land area in South Asia is used for agriculture, with over 40% of the population employed in the sector^2,3^. A large proportion of agricultural production in the region occurs during the southwest summer monsoon season^4^, with most of the region receiving over 80% of their annual rainfall during this time^5,6^. Rice is one of the most commonly grown crops, with India accounting for over 20% of global rice production, the majority of which occurs during the monsoon season^7^. Agricultural outcomes are closely connected to the monsoon. For example, in India a 1% change in annual monsoon rainfall causes a 0.34% change in India’s annual agricultural gross domestic product (GDP)^8^, a significant impact in a country where total agricultural production accounts for almost 18% of GDP^7,9,10^.

Rice production outcomes are known to be strongly linked to monsoon weather conditions (e.g. total rainfall^11^) and their temporal distribution (e.g. number of rainy days^12^). Changes in monsoon weather dynamics and variability therefore have important implications for rice productivity in India, for which hundreds of millions depend for livelihoods and food security. Past research has shown that the Indian monsoon has shifted towards a pattern of reduced total precipitation and less frequent rainfall events over the second half of the twentieth century, resulting in higher risk of both flood and drought damage^13–15^. Historic changes in monsoon dynamics in South Asia have been attributed to several factors, including increased aerosol concentration, land use changes and urbanisation effects^16,17^. In the future, climate change is likely to further exacerbate monsoon variability and extremes^18^. For example, studies suggest that the monsoon will arrive earlier and retreat later^19^, and with increasing annual variability and more frequent extreme precipitation events^20,21^.

To support adaptation of rice production to current and future monsoon weather variability, it is critical to understand which aspects of monsoon climate have greatest impacts on agricultural production outcomes. Recent modelling studies have found a range of weather factors to be important in explaining yield variability for rice and other crops in monsoon-dependent agricultural systems. For example, the importance of total precipitation has been well demonstrated^11^, with higher seasonal rainfall leading to higher rice yields. Similarly, a more even distribution of rainfall throughout the season has been linked with higher rice yields^12,22^, with extreme weather events such as droughts and high temperatures negatively affecting rice yields^23,24^. However, important gaps remain in our understanding about the effect of monsoon season length and onset timing on crop production^13,25^. For example, delayed monsoon onset and reduced season length have been shown theoretically^25–27^ and anecdotally^28,29^ to be potentially important drivers of crop yield losses, but are rarely included in statistical empirical crop-climate models in India and South Asia. Furthermore, past crop-climate studies in India and elsewhere exclusively focus on monsoon weather impacts on per-area crop yields, ignoring potential impacts on crop production areas^30^. Yet, emerging evidence suggests that changes to cropped area may also be an important outcome of weather extremes in climate vulnerable farming systems^31^. Failure to consider impacts of weather on crop harvested areas may therefore lead to an underestimation of the impacts of weather variations on agriculture and associated rural economies, with implications for the ability to anticipate future risks posed by climate change.

Relationships between weather and agricultural productivity are complex and non-linear, with production impacts dependent on the magnitude, sequencing, and timing of different weather extremes during the growing season^32–34^. In this context, a further key limitation of previous empirical modelling studies is the reliance on parametric regression approaches for linking production outcomes to observed weather variability. Parametric regression models assume pre-specified relationships between predictors and response variables, often specified as simple linear or quadratic relationships. This limits the ability to detect more complex or non-linear responses of crop yields to different aspects of monsoon weather, which have shown to be important for correctly attributing drivers of yield variability^35–37^. Machine learning (ML) methods, such as Random Forest (RF) models, offer a potential solution to this challenge through their ability to explore relationships between weather predictors and agricultural production outcomes without the need to pre-define the functional form of these relationships. Research has shown that RF models can significantly outperform parametric regression models in terms of ability to explain weather-yield relationships^35,38^. Moreover, RF models are capable of capturing key drivers and disentangling complex non-linear interaction effects^23,39^, and thus have significant potential to enhance understanding of weather-related production variability in monsoon-dependent agricultural systems in South Asia and elsewhere.

In this paper, we use RF modelling techniques in conjunction with an extensive long-term dataset of production and weather data from across four states in India (Punjab, Haryana, Uttar Pradesh and Bihar—collectively comprising the Indo-Gangetic Plains or IGP) to assess the dominant monsoon weather-related drivers of variability in rice yields and production area. Our findings provide comprehensive insights about the critical monsoon climate signals, and their non-linear interactions, that explain historic variation in rice yield and production area across the IGP, the latter of which has rarely been considered in past crop-climate impact studies. In doing so, our findings provide key evidence to strengthen understanding of how future changes in monsoon dynamics and variability may impact rice production in India, and help to guide efforts to enhance climate resilience of agriculture and rural livelihoods across South Asia.

## Data

### Study region

**Figure 1:**
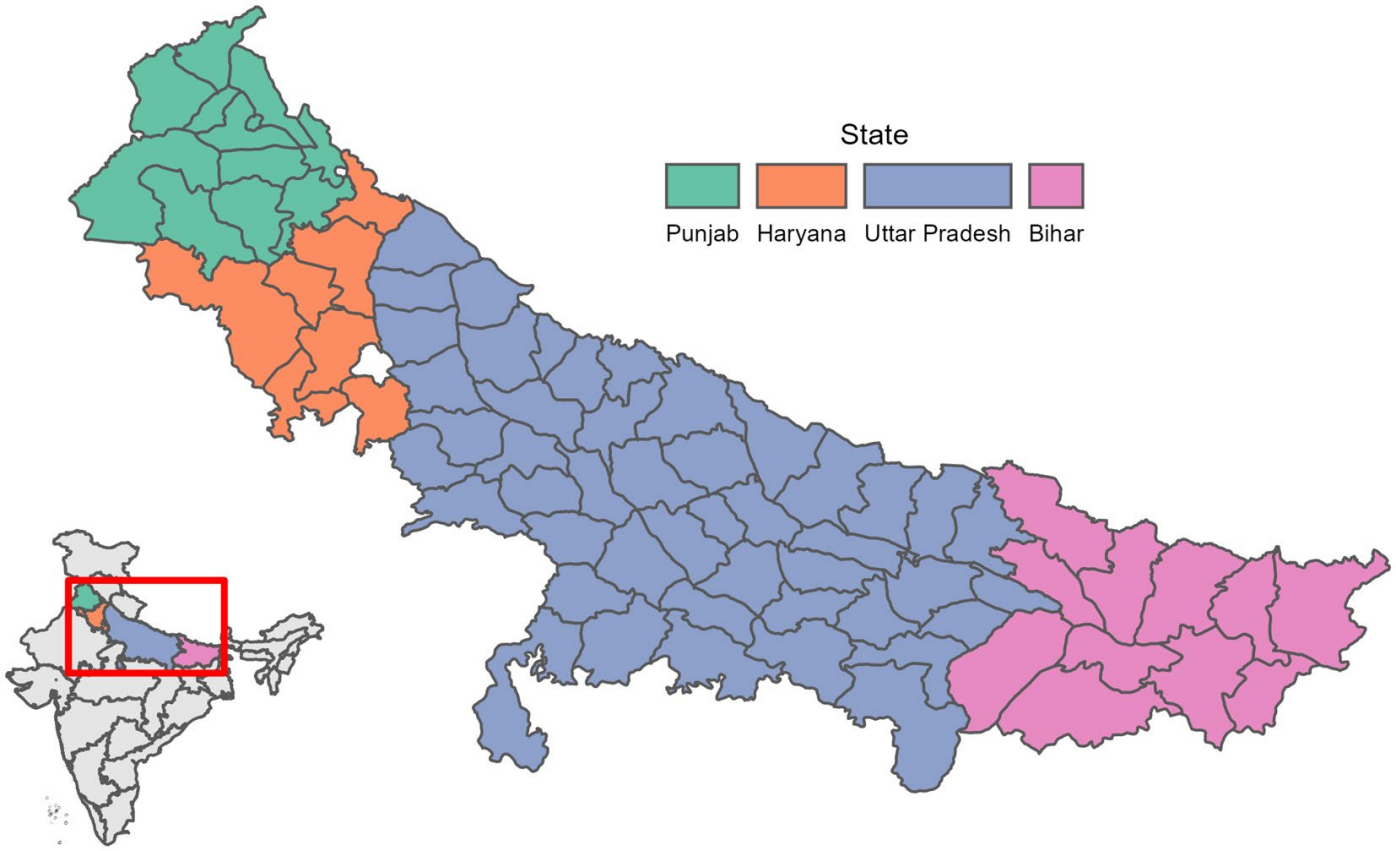
Colours represent the four states of the IGP in this study. Further divisions by black lines represent 1966 district boundaries. Districts in grey have been removed from analysis due to their urban or mountainous geography (see supplementary information).

Our study focuses on the IGP, which for the purposes of this study is defined as including 71 total districts within 4 states (according to 1966 boundaries from DLD): Punjab, Haryana, Uttar Pradesh and Bihar (Fig. 1). Agricultural production in the IGP is characterised primarily by a rice-wheat crop rotation, with rice cultivated during the Kharif (monsoon, May to September) season and wheat during the Rabi (dry, November to April) season^40^. The IGP is commonly referred to as the ‘breadbasket of India’ because it produces 40% and 70% of India’s total rice and wheat output respectively^41^, and is therefore vital to both national and regional food security. Temporal variability in rice productivity across the IGP is strongly linked with the variability of the Asian summer monsoon, which has large inter-annual variability^42,43^. Thus, the IGP represents an ideal study area to explore links between rice production outcomes and monsoon weather conditions.

### Agricultural production data

District-level data on rice yields and harvested production areas for the 71 administrative districts in the four states of the IGP was obtained from ICRISAT’s District-Level Database (DLD)^44^. The dataset provides information on yields, harvested area and production for a 52-year period (1966–2017) and has been used in a number of previous studies of yield variability in India, for rice and other crops^24,45,46^. These data have been sourced from quality-controlled state and district surveys and reports collated by the Directorate of Economics and Statistics of the Government of India and made available by ICRISAT. The DLD data was chosen because it provides a robust and trustworthy official record of observed agricultural production, with sufficient spatial resolution (district-level) and temporal extent to enable exploration of climatic drivers of rice yield and production variability. Production data for the four states of the IGP were filtered to remove districts where rice production was minimal (Fig. 1, shaded grey) so as to avoid these data having undue influence on subsequent analyses (see supplementary information).

A large proportion of historic rice yield and production variability in the IGP, and indeed India more broadly, is attributable to non-weather related temporal and spatial trends. For example, rice yields across India have increased over time due to progressive improvements in technologies, new crop varieties, and CO_2_ fertilisation^47^. Persistent spatial differences also exist in rice yields and production outcomes between districts and states due to varying levels of agricultural intensification, mechanisation and land suitability. We remove such obfuscating trends in production data prior to running the model using state-specific locally weighted scatterplot smoothing (LOWESS) curves (Supplementary Fig. S2). We remove trends at the state and not district level to avoid overfitting; this is consistent with previous studies^13,25,32^.

### Weather data

To calculate values of each weather variable for each district and year of our agricultural production record, we use the Princeton Global Forcing (PGF) gridded weather dataset^48^. PGF was selected due to its long timespan and relatively high spatial resolution (0.25°x 0.25°), and has been used by other crop-climate modelling studies^49–51^. PGF has also been validated for use over India, specifically including the temperature^52^ and precipitation^53^ variables used in this study. Furthermore, rice yield sensitivity to the PGF weather variables matched that of similar weather datasets in an India-based comparison^54^. Values of each weather variable included in the modelling were aggregated spatially to district-scale using weighted means with DLD boundaries, and temporally to growing-season-scale (1st May to 30th September).

**Table 1:** Overview of predictor variables in the random forest models.

Variable (units)	Description (references)
Total precipitation (mm)	Total growing season precipitation^12,55,56^
Proportion of rainy days	Proportion of growing season days where rainfall is above 2.5 mm, the Indian Meteorological Department’s definition of light rain^12^
Growing degree days	A measure of heat accumulation (time temperature is above 10 °C) over the growing season^57^
Mean minimum temperature (°C)	Mean daily minimum temperature over the growing season^55,58^
Mean maximum temperature (°C)	Mean daily maximum temperature over the growing season^55,58^
Downwelling shortwave radiation (W/m^2^)	Sum of DSWRF over the growing season^25^
Onset deviation (days)	Deviation of monsoon onset from the all-year district mean onset^59^
Monsoon season length (days)	Number of days between monsoon onset and demise^59^
Irrigated rice area proportion	Irrigated proportion of total rice area per district^60^

All variables have been aggregated from gridcell (0.25°x 0.25°) to district scale by weighted means. Yield data has been detrended and normalised prior to use in the model and therefore no spatial or temporal trend terms are included here. Growing season is defined as 1st May to 30th September.

The goal of our study is to understand which aspects of monsoon weather have greatest impacts on rice production variability in the IGP, both individually and in combination. The types of variables included in ML models greatly affect the output and performance of the model. A full list detailing weather variables included in our modelling can be found in Table 1. Basic measures of temperature (aggregated growing degree days, daily minimum and maximum temperatures) and precipitation (total precipitation, proportion of rainy days) were selected due to their importance in many past weather-crop modelling studies^23,39,57,61–63^. In addition, we include several further monsoon weather variables that have largely been neglected in past studies: (1) Downwelling shortave radiation flux (DSWRF), a measure of incoming surface-level solar radiation affected by frequent cloud cover in South Asia^25,64–66^; (2) Monsoon season length, and (3) Monsoon ‘onset deviation’, both of which can critically affect sowing, transplanting and harvesting efficacy^45,59,67,68^. For additional details on the calculations of monsoon variables, see ‘Weather data’ section of the supplementary information.

## Methods

We used the RF algorithm to quantify the relationships between monsoon weather variables and rice production across the IGP over the period 1966 to 2016. RF models are non-parametric statistical models that work by aggregating the outputs from an ensemble of decision trees. We use a regression RF model where the model output is the mean of each tree’s out-of-bag (OOB) output^69^. This relatively simple structure makes RF modelling an ideal method because it enables evaluation of complex non-linear relationships and interactions created during model construction, whilst retaining model transparency that is sometimes lost in other ML approaches. Support vector machine (SVM) modelling was also considered for use in this study but was discarded due to the improved interpretability of relationships formed within RF models. SVM models have often performed similarly to RF models (see review^70^), but lack outputs that enable transparent interpretation of relationships formed within the model^71–73^.

In particular, ML is useful for crop-climate modelling where there are many non-linearities and interactions between weather predictors and production outcomes that would be challenging to comprehensively specify *a priori* in a traditional parametric model structure. Similar studies have used RF modelling previously to detect and interpret complex non-linear relationships between predictors and response variables, including in crop-climate modelling^23,55,74^.

We develop two separate RF models quantifying relationships between monsoon weather variability and detrended anomalies in margins of production: (1) rice yields (intensive), and (2) harvested rice areas (extensive). Hyperparameter tuning and model runs were performed using ‘tidymodels’ R package v0.1.3 and ‘randomForest’ R package v4.6-14 (mtry = 3, min_n = 5, ntrees = 500) in R v4.0.4^69,75,76^. We evaluate the accuracy of each RF model based on the mean OOB root mean square error (RMSE) rate. Relative importance of each predictor variable within each RF model was assessed using ‘%IncMSE’ (aka ‘permutation’) as the measure of importance (see supplementary information), which was used to create variable importance plots (VIP). Because VIPs are affected by the internal splitting of training data and random selections in a RF model, we ran 100 RF models with different seeds. This was done to provide 95th percentile ranges, representing the variability of model output^77^. The same 100 RF model outputs were also used to calculate mean percentage of explained variance and RMSE for models (1) and (2).

Accumulated local effects (ALE) plots were used to visualise the influence on production outcomes of each predictor, including any non-linearities in responses. For examination of main effects, ALEs were selected over typical partial dependence (PD) plots because they more reliably account for interactions between predictors^60,78–80^. Similar to VIPs, ALE plots are affected by the split of training data. Therefore, results are presented as 95th percentile ranges from 100 RF model runs. The interpretation of second-order ALE plots is difficult and cumbersome, requiring simultaneous assessment of the main-effect ALE plots. Therefore, PD plots were used to assess second-order interactions because they include interaction effects, making for more straightforward interpretation. All pairwise interactions were plotted, but only those pairwise interactions of substantial effect are used for analysis.

## Results

### Model performance

**Figure 2:**
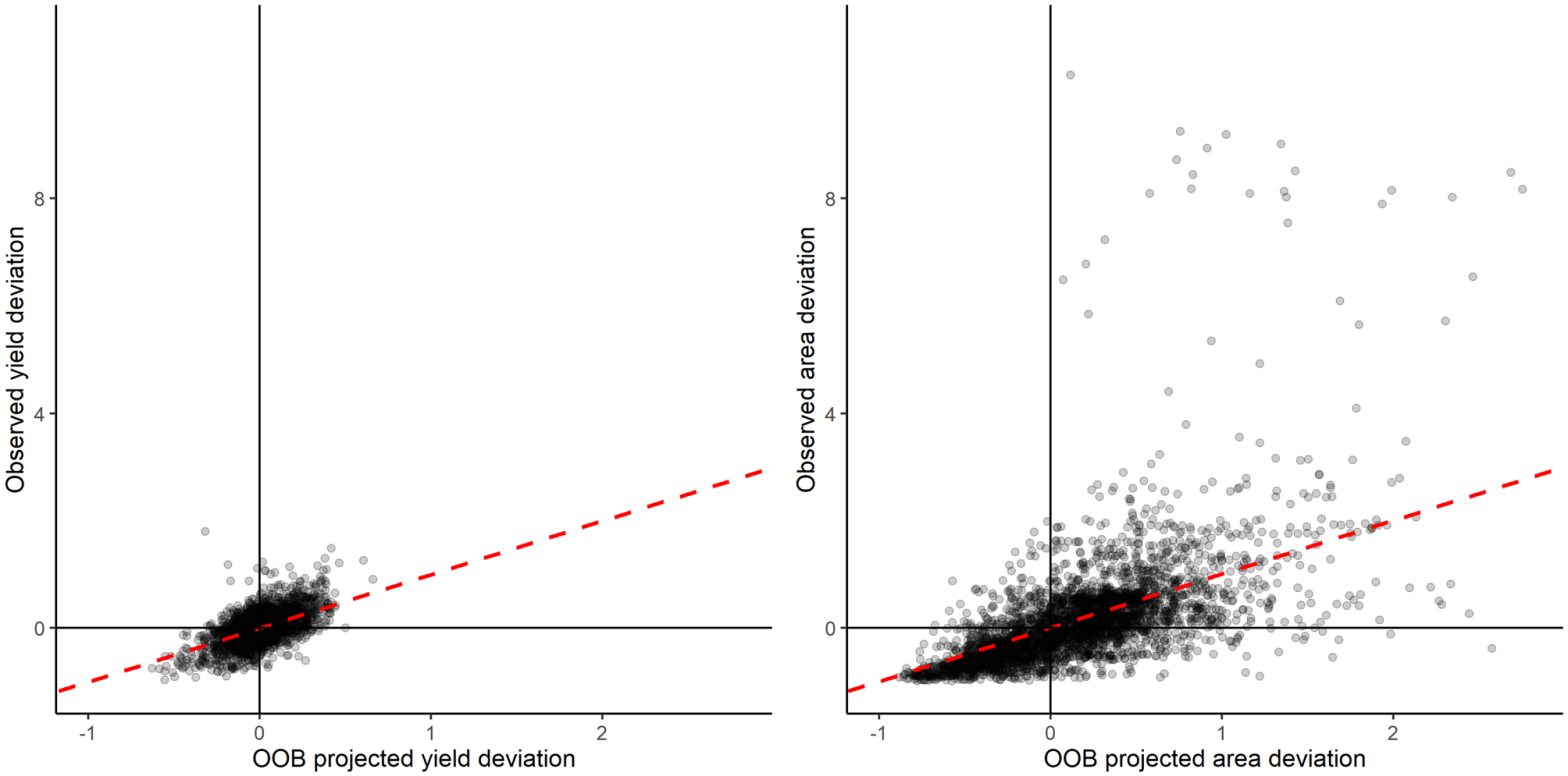
Random forest model projected values with observed yield and area anomalies. Red dashed lines have intercepts of 0 and slopes of 1.

Weather predictors in the RF models were able to explain similar levels of OOB anomaly variation in both rice yield (33%, RMSE = 0.215) and area harvested (35%, RMSE = 0.792). Furthermore, OOB anomalies estimated by RF models reliably captured instances of both above and below average yield (69% agreement) and area (77% agreement, Fig. 2). When temporal trends and spatial fixed effects terms that were previously removed were added back into the RF models, the amount of OOB variability explained by the models increased to 87% (yield) and 73% (area), comparable to previous fixed and random effects parametric models of crop-climate relationships in India and other smallholder farming systems^11–13,81^. These results highlight the important role that trends in agricultural outcomes over time (e.g. technology or variety improvements) and space (e.g. persistently higher yields in some states) have in explaining the overall variations in outcomes across our sample dataset, while also demonstrating the ability of RF models to explain a meaningful proportion of remaining weather-related variance in agricultural outputs.

### Yield response model

**Figure 3:**
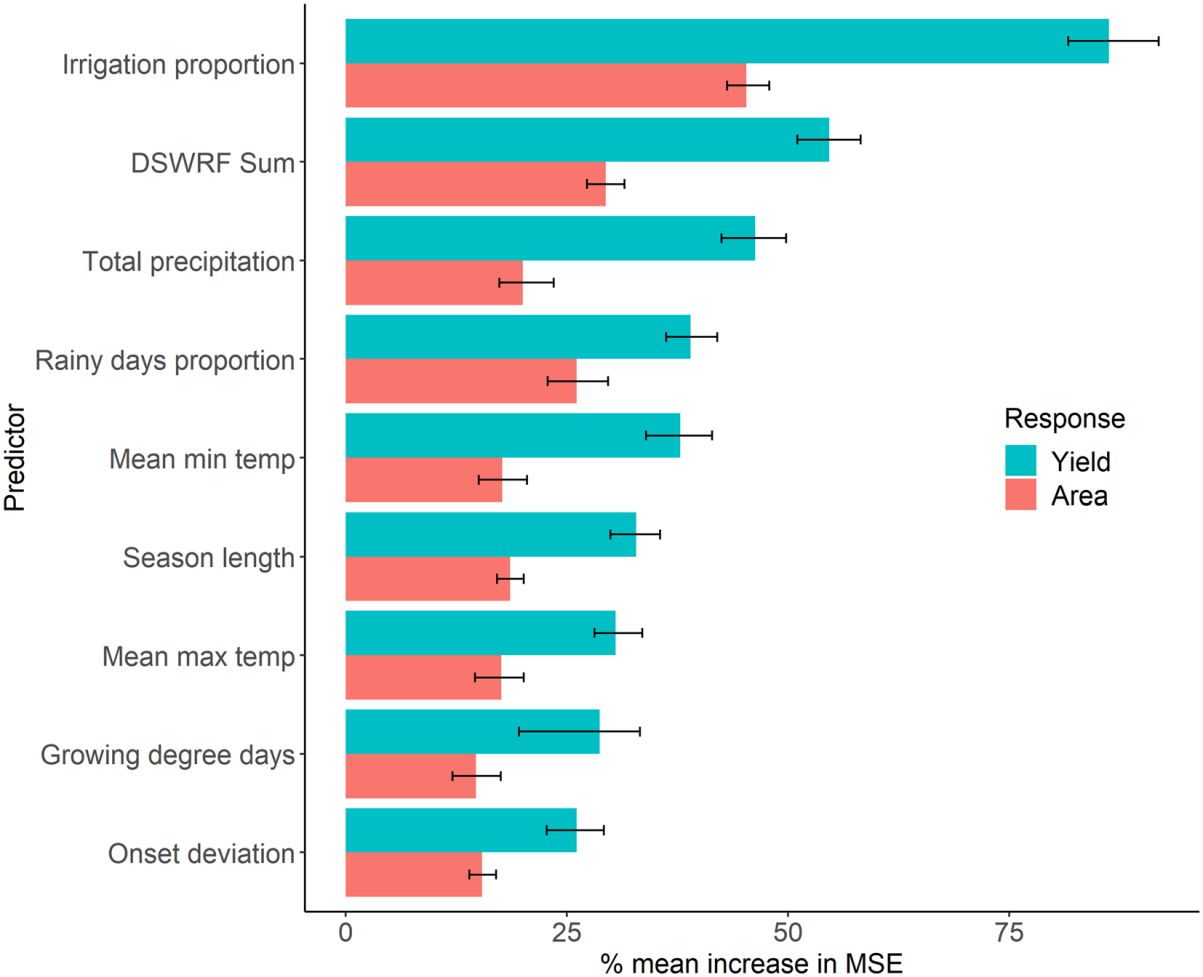
Variable importance plot from the random forest models calculated as percentage increase in mean-square error (MSE). Main bars represent mean value from 100 runs, inset whiskers represent 95th percentile of results.

The proportion of rice cropped area under irrigation within each district was the most important predictor of rice yield anomalies in our RF model (Fig. 3). This was expected due to the dependence of rice yield on water availability, and given the heterogeneous levels of access to irrigation over space and time in the IGP. The most important weather-related predictor of rice yield anomalies was downwelling shortwave radiation flux (DSWRF, Fig. 3). Permutation of the seasonal sum of DSWRF values caused a 55% increase in MSE, notably larger than for other monsoon weather characteristics. Total precipitation and proportion of rainy days were the next most important drivers of rice yield anomalies, reflecting rice’s large water requirements and vulnerability to water-related extremes, especially in the absence of accessible supplemental irrigation. Remaining aspects of monsoon variability, which include a range of water and temperature related variables, have similar variable importance scores, with a marginally higher ranking for mean minimum temperature during the growing season. Permutation of any one of these variables results in a meaningful change in %MSE of the model (> 25%), highlighting the complexity and diversity of mechanisms through which rice yields are impacted by monsoon weather variability.

**Figure 4:**
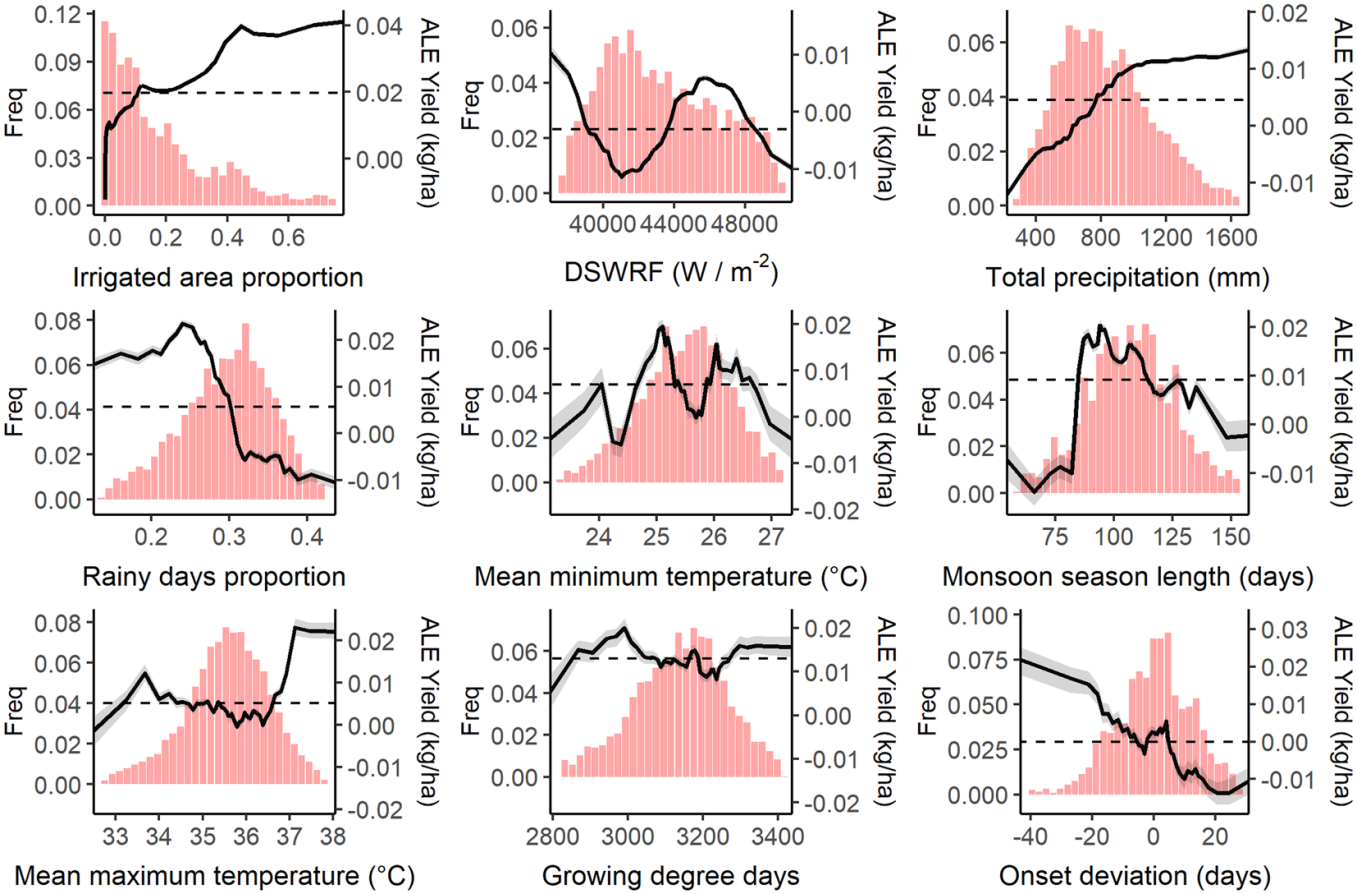
Accumulated Local Effects (ALE) plots of the yield anomaly response model for each predictor variable. 100 random forest models were run with different seeds, solid black lines represent mean ALE and ribbons represent 95th percentile ALE. Histograms show the distribution of the 98th percentile of predictor data in the original full dataset prior to sub-sampling for the random forest modelling. Note that y-axis ALE values are contingent on input data and are not directly comparable. Y axis scale varies between each sub-plot to highlight response curve shapes.

**Figure 5:**
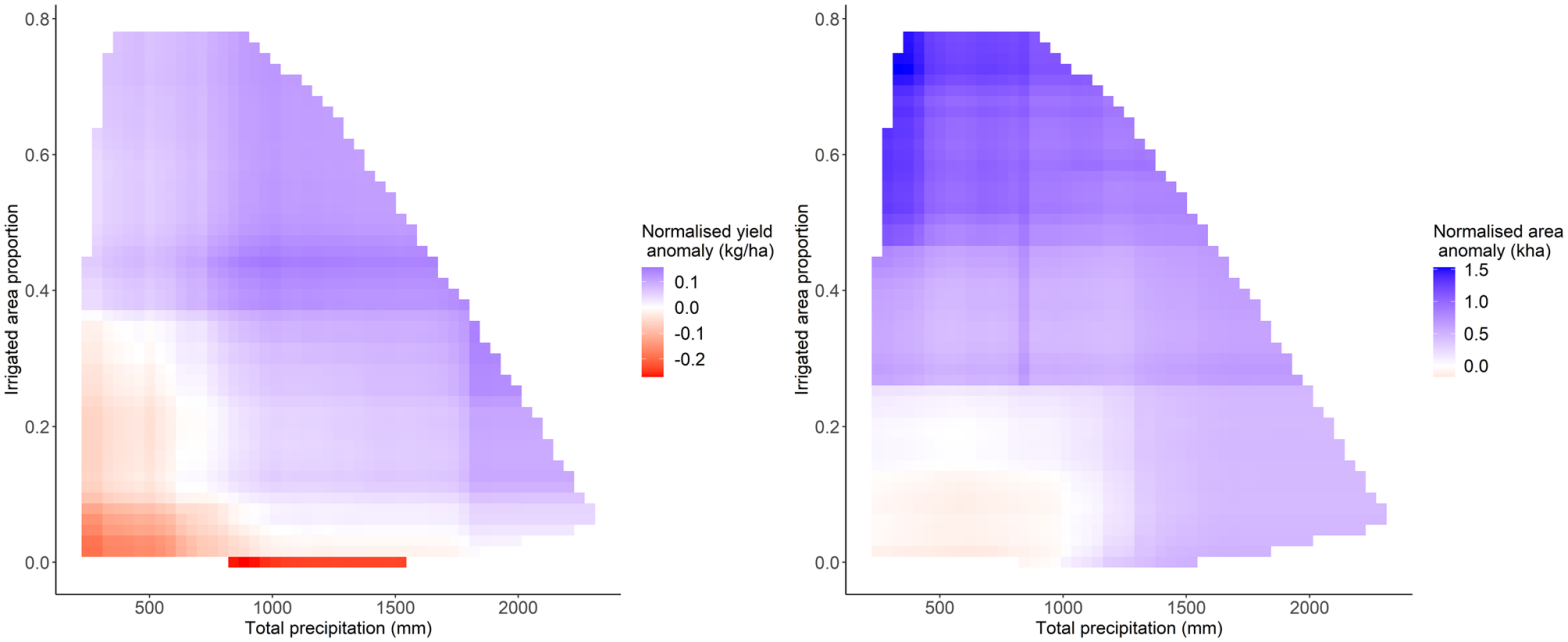
Partial dependence two-way interaction plots for the yield and harvested area response models.

Our analysis identified several monsoon characteristics that were strongly associated with positive rice yield anomalies. Above a DSWRF threshold of approximately 43,500 W/m^2^, the positive marginal effect of DSWRF on rice yields increased until around 45,500 W/m^2^ (Fig. 4). Positive yield anomalies were also observed for higher total seasonal precipitation above a threshold of 800 mm, beyond which the positive marginal effect increases up to 1000 mm where the effect then plateaus. Season lengths between 80 and 115 days were further found to be an important predictor of positive yield anomalies. A strong interaction effect was detected between the proportion of irrigated area and total precipitation measures (Fig. 5). In particular, high precipitation is linked with positive yield anomalies even when irrigated areas are low (and vice versa), reflecting the fact that irrigation is used to supplement rather than replace rainfall for rice cultivated during the monsoon season.

At other extremes of their observed ranges (e.g. DSWRF below 43,500 W/m^2^, total precipitation below 800 mm, season lengths below 80 and above 115 days), these variables were instead found to be associated with negative yield anomalies, reflecting the sensitivity of rice development to appropriate radiation and water-related conditions. Several additional monsoon characteristics were found to be important predictors of negative yield anomalies despite having lower overall variable importance in our RF models. For example, rice yield anomaly ALE values become negative as monsoon onset is delayed by five days or more relative to the average expected onset date for a given district. Additionally, yield anomalies switched sharply from positive to negative when the proportion of rainy days exceeded 30% (Fig. 4). This unexpected negative effect of well-distributed seasonal precipitation^12^ may be due to the correlation between rainy days proportion and total precipitation (Supplementary Fig. S4).

### Area response model

**Figure 6:**
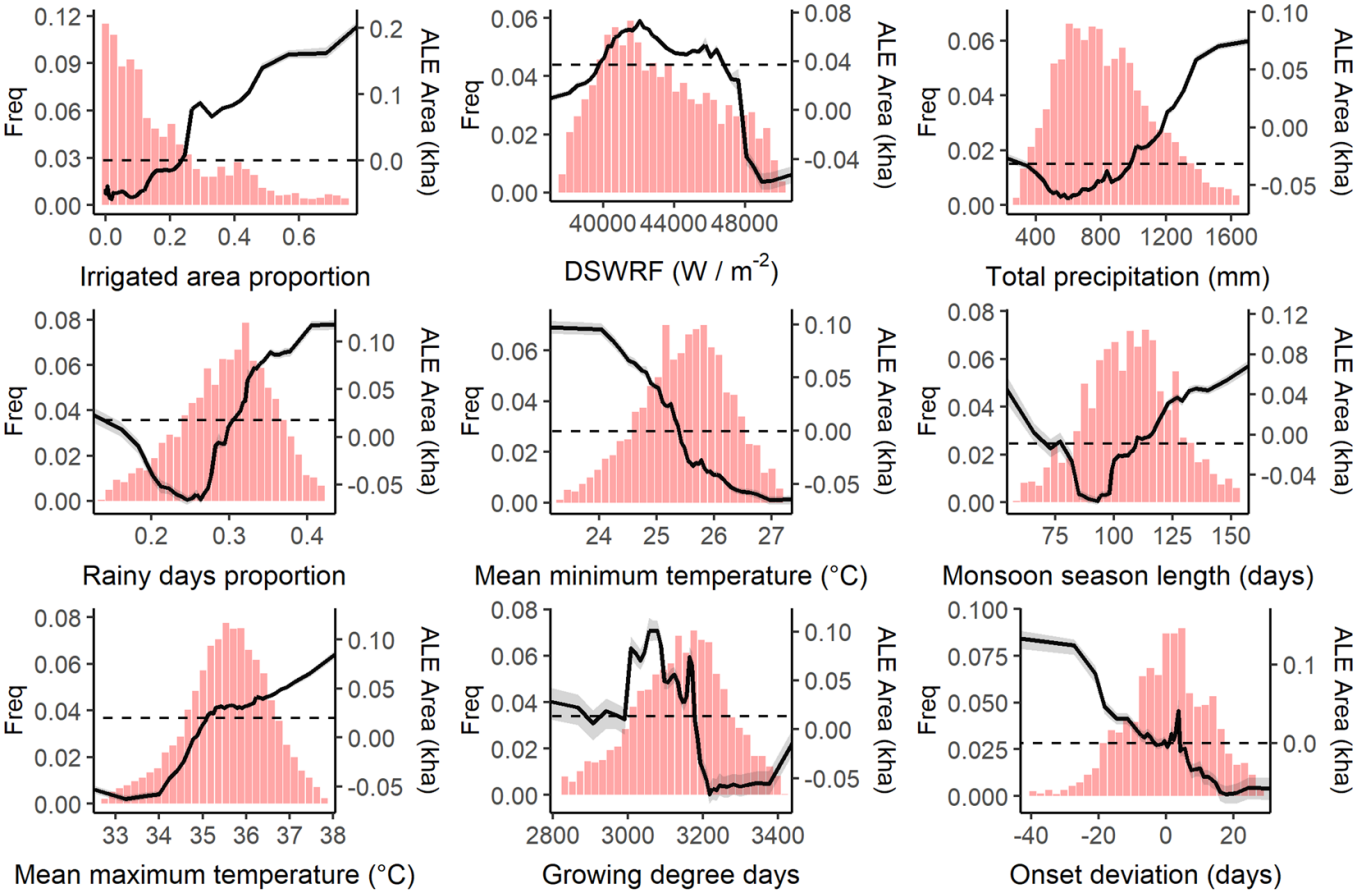
Accumulated Local Effects (ALE) plots of the harvested area anomaly response model for each predictor variable. 100 random forest models were run with different seeds, solid black lines represent mean ALE and ribbons represent 95th percentile ALE. Histograms show the distribution of the 98th percentile of predictor data in the original full dataset prior to sub-sampling for the random forest modelling. Note that y-axis ALE values are contingent on input data and are not directly comparable. Y axis scale varies between each sub-plot to highlight response curve shapes.

The response of harvested area anomalies (Fig. 6) closely matched that of yield anomalies for irrigated area, total precipitation, maximum temperature and monsoon onset deviation predictors. For example, the ALE effect of onset deviation on area harvested switched from positive to negative for onset delayed longer than three days, close to the five-day delay threshold of the yield response model. Similarly, greater total precipitation leads to higher positive marginal effect on area anomaly, but only above a threshold of 1000 mm compared to the 800 mm threshold for yield anomalies. The same interaction effect pattern detected between total precipitation and irrigated area in the yield model was also detected in the area anomaly model (Fig. 5).

Area anomaly responses differed from those found previously for yield anomalies for two of our predictor variables, the proportion of rainy days and monsoon season length. The marginal effect of increasing the proportion of rainy days becomes increasingly positive above a threshold of 30%. Greater monsoon season lengths generally had a positive impact on rice harvested areas, with exception of a small negative effect for season lengths between approximately 75 and 110 days. In addition, the response of area anomalies to minimum temperature exhibited a more distinct negative linear trend in contrast to the mixed signal in the yield anomaly response.

## Discussion

Our study leverages data-driven machine learning approaches to deepen understanding of the relationship between rice production outcomes and climate variability in monsoon-dependent agricultural systems in South Asia. Our results show that RF machine learning models are capable of identifying and attributing historical relationships between weather and rice production variability in India, consistent with studies for other crops and in different regions^35,82^. Our RF model simulations capture the complex non-linear responses of rice production to interacting characteristics of the South Asian monsoon, providing novel insights about non-linear crop-climate responses and variable interactions that would be challenging to identify in traditional parametric regression analyses^57,83,84^. Critically, our findings demonstrate the important role that adjustments to harvested area play in determining the overall impact of monsoon weather shocks on rice production outputs. This is an important and novel result because harvested area responses have largely been neglected in previous crop-climate studies in the region—both using statistical and process-based models—that focus solely on impacts of weather variability on per-area yields^85–87^.

In line with previous studies, our findings support the conclusion that total seasonal precipitation and irrigated area explain a significant proportion of weather-related rice yield variability in India and South Asia more widely^11,13,88,89^. The high relative importance of irrigation reflects the heterogeneity in access to and use of irrigation in space and time across the IGP^90^. Lower levels of irrigation in some district-years limit farmers’ abilities to buffer rice against water and heat-related weather extremes (Fig. 5). Beyond a seasonal precipitation threshold of around 1,000 mm, the positive marginal effect of increased precipitation is reduced significantly. This type of precipitation insensitivity threshold has been found previously in other regions, for example for maize and sorghum in sub-Saharan Africa and North America^39,55^ as well as for rice in China^91^. The importance of solar radiation as a driver of yield outcomes in this study was also consistent with past studies in other regions^58,92^, although it is notable that solar radiation has often been neglected as a driver of crop yield variability in previous studies in India and South Asia^11,93,94^.

A key novel finding from our analysis is that negative yield and harvested area anomalies occur when the monsoon onset is late by more than a few days, relative to expected local onset timings. This finding is likely to reflect the negative impact of delayed transplantation on rice yields and production. Delayed transplantation occurs when the monsoon is delayed and irrigation is absent or underutilised^45^, meaning that there is insufficient water to move plants from the nursery into the main fields. This delay in transplanting leads to rice plants being transplanted at an older age, a factor that is associated with reductions in productivity due to combined impacts of a larger transplantation shock and greater vulnerability to cold stress later in the season^45^. Given the increasing variation in monsoon onset, demise, and length^18,27,95,96^, failure to account explicitly for these factors in crop-climate models may lead to errors in the estimation of yield extremes (including losses), in particular when models are used to project impacts of increasingly variable future climate scenarios. Through exploration of interaction effects, we show that negative effects from a delayed onset can be mitigated through increasing irrigation, suggesting importance of expanding irrigation to ensure timely crop establishment and buffer against dry spells. This matches expectations based on previous studies, that yield losses from a delayed onset are often associated with intraseasonal dry spells^67,97,98^.

Our findings also highlight the importance of a number of additional monsoon characteristics in explaining rice production variability, which have not been included in prior crop-climate studies for rice and other crops in South Asia. Monsoon season length, for example, is not commonly included as a predictor variable in modelling studies, with weather variables instead computed over arbitrary fixed calendar windows^12,22,81,88^. We find that monsoon season length is an important determinant of both area and yield anomalies, and that its effects are bounded by two distinct thresholds of season length (75 and 115 days). However, the response of monsoon season length may be complicated by non-weather factors and interactions. For example, a longer growing season may be beneficial for longer duration rice varieties, but may also increase risks of the crop being impacted by erratic or extreme rainfall at the time of harvest.

We note that the negative yield effect of a greater proportion of rainy days found in this study is contrary to expectations. Generally, a greater proportion of rainy days would be expected to reduce the risk of crop yield losses due to dry spells and associated plant water stress^12^. At the same time, the consistently positive relationship between total precipitation and rice yield and area anomalies found in this study implies that even at high levels of precipitation, yield and harvested area are not negatively affected, as might be expected from flooding or lodging damage^99^. However, these two unexpected relationships (total precipitation and rainy days with yield anomalies) may be explained by a mixed precipitation signal within the model. The proportion of rainy days data is positively correlated with total precipitation data (0.57, Supplementary Fig. S4). High values of rainy days may therefore represent an excess rainfall signal, where submergence and physical flood damage reduce yield, in particular if occurring during critical crop development periods.

While offering a powerful tool for crop-climate analysis, it is important to acknowledge that the non-linear relationships formed within RF models may also pose challenges for interpretation and identification as demonstrated in parts of our results. For both temperature- and precipitation-derived predictors, the weather signal may be split unevenly and unpredictably between multiple variables. For example, the irrigated area, total precipitation, rainy days proportion, onset deviation and season length variables were all selected to capture different aspects of the precipitation signal. The many complex interactions between these variables within the model may obfuscate the true precipitation signal for each individual predictor. This may also explain the unexpected impacts of rainy days found in our analysis, for which a positive effect on yields was expected^11,12^. Similarly, using temporal aggregations of weather variables over the monsoon season also limits the precision of response curves that can be derived using RF modelling. Future work should seek to explore the benefits of considering the intraseasonal distribution and sequencing of weather variables in order to improve the ability of RF models to disentangle monsoon crop-climate relationships and to support in-season forecasting of crop production.

District scale data used in our study reveals broad patterns in the response of rice production outcomes to monsoon weather predictors. However, our conclusions are limited by relatively coarse spatial resolution of the rice production data and accompanying weather data used as inputs to our RF models. Aggregation of data to the district scale is likely to obscure important local heterogeneity in agricultural productivity, potentially resulting in an underestimation of the true impacts of spatially heterogeneous weather-related extremes on crop yields and harvested areas^100^. For example, our RF model reveals a negative impact of delayed monsoon onset on rice yield, but this effect may be higher if it were possible to account for sub-district level heterogeneity in rainfall and associated rice yield outcomes.

## Conclusions

Our study contributes to the growing body of literature that seeks to identify and assess impacts of climate variability and change on agricultural production outcomes in smallholder farming systems^101–103^. Our findings demonstrate the capability of RF machine learning models to capture and explain variability in rice production outcomes in response to monsoon climate signals. Critically, we demonstrate the additional impacts of monsoon weather variability on crop production area, highlighting the importance of considering changes in both per-area crop yields and harvested areas when assessing impacts of weather on agricultural production. Our modelling identifies complex non-linear yield and harvested area response signals from weather variables which can be used to better guide future efforts to support adaptation of rice production to extreme weather and climate change. In particular, we demonstrate the importance of considering additional characteristics of monsoon variability beyond standard measures of precipitation and temperature, including variations in the onset and duration of the monsoon which are expected to be impacted significantly by climate change in the region^21^. More broadly, our findings reaffirm the importance of irrigation access as a critical buffer against monsoon variability, both historically and under future climate change. In this context, there are urgent needs to intensify and expand access to water in areas of the IGP with currently low levels of irrigation use (e.g. Bihar), while also simultaneously implementing measures to improve long-term sustainability of water management in other parts of the region (e.g. NW India) to mitigate future negative impacts on agricultural productivity and climate risk^104^.

## Supplementary Information

Supplementary Information.

## Data Availability

Crop production data supporting this publication are freely available from ICRISAT’s district-level database, http://data.icrisat.org/dld/src/crops.html^44^. Raw weather data is available from the Terrestrial Hydrology research group at Princeton University, http://hydrology.princeton.edu/data.pgf.php^48^. Sample data and code are available on fig share (https://figshare.com/projects/Identifying_links_between_monsoon_variability_and_rice_production_in_India_through_machine_learning/151362) with further information available upon reasonable request from the authors.
