# Supplementary material for: Identifying links between monsoon variability and rice production in India through machine learning
Source: Sci Rep. 2023 Feb 10;13:2446. doi: 10.1038/s41598-023-27752-8 (PMC9918484; doi:10.1038/s41598-023-27752-8)
Supplement: Supplementary file 1 — Supplementary Information. [file 41598_2023_27752_MOESM1_ESM.pdf]

# Supplementary Information

## Identifying links between monsoon variability and rice production in India through machine learning

**Christopher Bowden[1,*], Timothy Foster[1], and Ben Parkes[1]**

[1]University of Manchester, Department of Mechanical, Aerospace & Civil Engineering, Manchester, M13 9PL, UK
[*]christopher.bowden@postgrad.manchester.ac.uk

## Production data

We applied a filter to remove districts with minimal rice production from the DLD dataset. Specifically, any district where rice production area was < 1 % of the total district land area or < 1000 ha in more than half (26) of the study years was removed from the dataset. This resulted in the removal of 6 out of 77 districts. The six districts removed were either urban areas (southern: Chandigarh, Delhi, Jalaun, Mahendragarh, Agra) or highly mountainous areas (northern: Hamirpur) with small agricultural production areas and were deemed not relevant to this study (Fig. 1, shaded grey).

Although the ICRISAT DLD does not distinguish between rice production in the two main growing seasons in India (Kharif and Rabi), for the states in the IGP, the overwhelming majority of rice is grown in the Kharif season and not double-cropped in the Rabi season[1,2]. The lack of growing season-specific yield information in the DLD will not limit the analysis, as reported rice yields and production areas relate predominantly to cultivation during the summer monsoon season. This is in contrast to areas of eastern India with assured rainfall and irrigation (e.g. Odisha and West Bengal), where two or more rice crops are cultivated each year, justifying our decision to focus analysis specifically on the four states of the IGP[3,4].

Two approaches exist to remove obfuscating temporal and spatial trends when seeking to identify the role of weather variability in observed crop yield and production outcomes: identifying and removing the trends from the response variable prior to running the model[5,6]; or including spatial and temporal control terms within the model[7–9]. Here, we adopt the latter approach to remove trends in yield data prior to running the model. State-specific LOcally WEighted Scatterplot Smoothing (LOWESS) curves were fitted to the yield data (Supplementary Fig. S2) and subtracted from each state's yield data, removing the temporal trends by state. Detrended yield values were then normalised by dividing by the same LOWESS curve values. Absolute yield values vary significantly across the IGP, so focusing on normalised yield anomalies allows inter-comparison of effects of weather variables across all districts in the region. Similar normalisation techniques have been used to successfully isolate weather-related yield variability in previous crop-climate modelling studies[8,10–12], supporting our use of this approach in this study.

## Weather data

Monsoon onset and demise were calculated from PGF precipitation data following a modified version of the cumulative precipitation anomaly method proposed by Noska and Misra[13]. Specifically, for each cell-year, we calculated the daily precipitation anomaly based on the difference between the daily precipitation rate and mean daily precipitation, and then fit a curve to the daily cumulative anomaly values over the annual time series ('lowess' function of R 'stats' package v3.6.3). The minimum and maximum cumulative anomaly for each cell-year were then taken as the onset and demise, respectively, of the monsoon (Supplementary Fig. S3). Each district-year's onset and demise were subsequently calculated from a weighted average of their constituent cells. This approach was selected as it provides a more objective and reliable method for estimating both onset and demise over a large spatial and temporal range compared to agronomic[14] or breakpoint[15] definitions. Aggregated Growing Degree Days (AGDD) were also computed prior to aggregation. For each cell-year, a sine curve was fitted between daily minimum and maximum temperatures. The GDD was then calculated as the area under the sine curve, bounded by a lower threshold of 10°C. Key details of other variable calculations are summarised in Supplementary Table 1.

## Statistical modelling

Tuning of hyperparameters was performed using the package 'tidymodels' v0.1.3[16]. As a result of tuning, the following hyperparameter values were used in all modelling runs: number of trees: 500, mtry: 3, minimum node size: 5.

‘%IncMSE’ was used to assess variable importance in the RF models. This measure represents the percentage increase in mean-square error (MSE) of the model as each predictor variable is iterated over its full range of values. For each variable permutation, those that result in greater changes in model MSE are ranked as more important[17].

During its calculations, a PD plot averages the response variable resulting in unrealistic data points in its surrogate models. On the other hand, ALE plots accumulate local effects and thus do not generate unrealistic data points. This makes ALE plots less prone to errors in the presence of strong interactions between predictors.

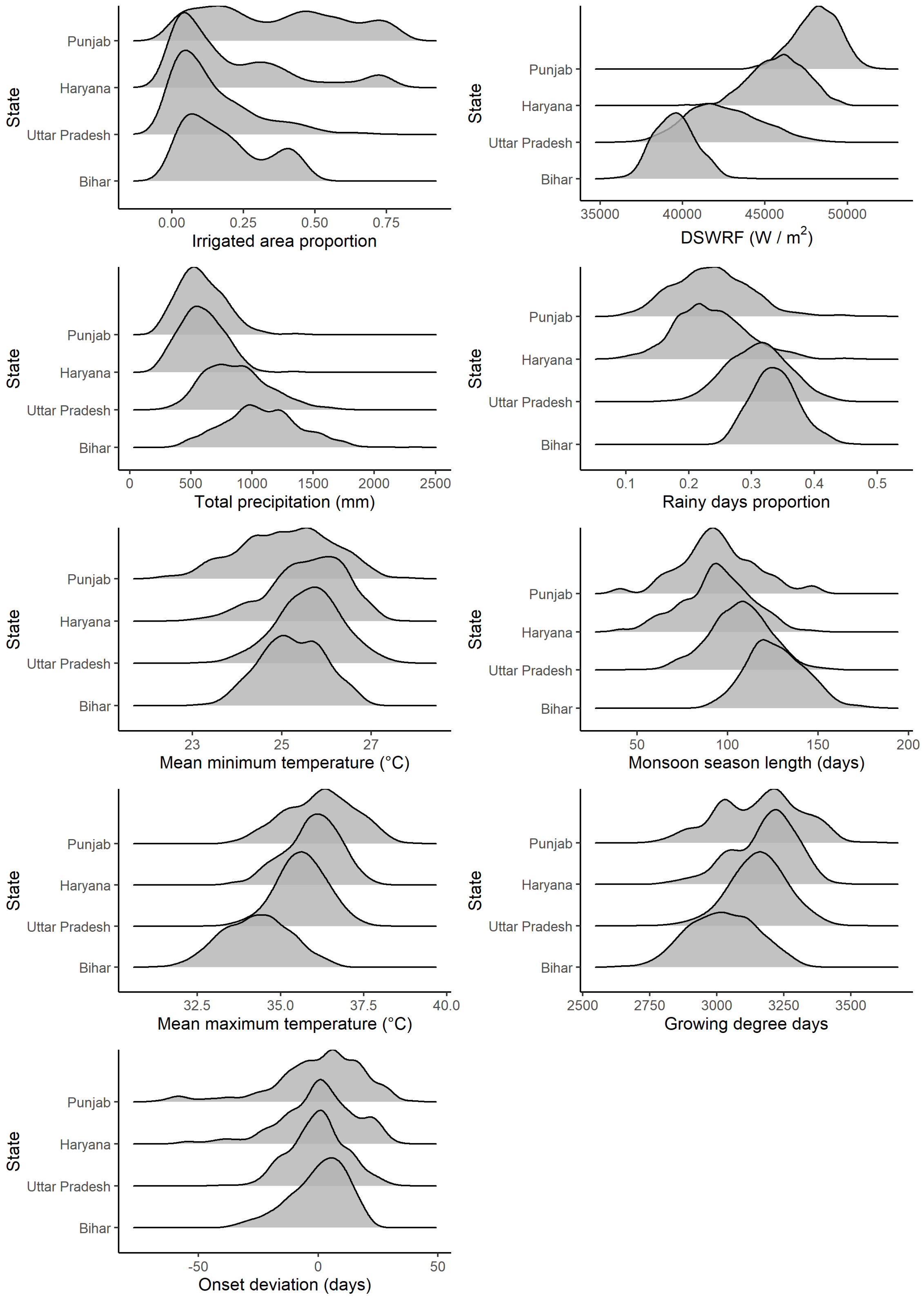


**Supplementary Figure S1.** Violin plots represent distribution of district-year values over 52 years for all predictor variables. Y axes read from top to bottom are states from West to East.

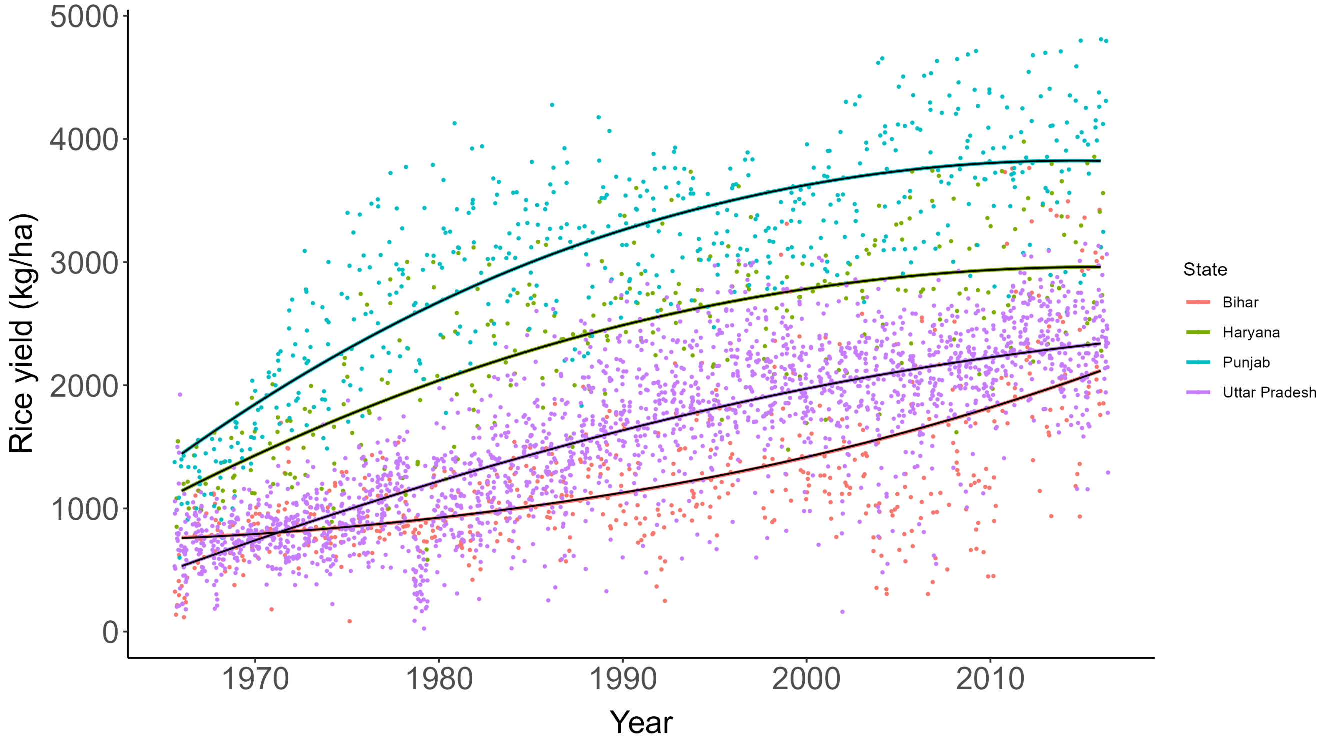


**Supplementary Figure S2.** Locally weighted scatterplot smoothing (LOWESS) curves (black) fitted to raw district-level yield data to remove temporal trends by state.

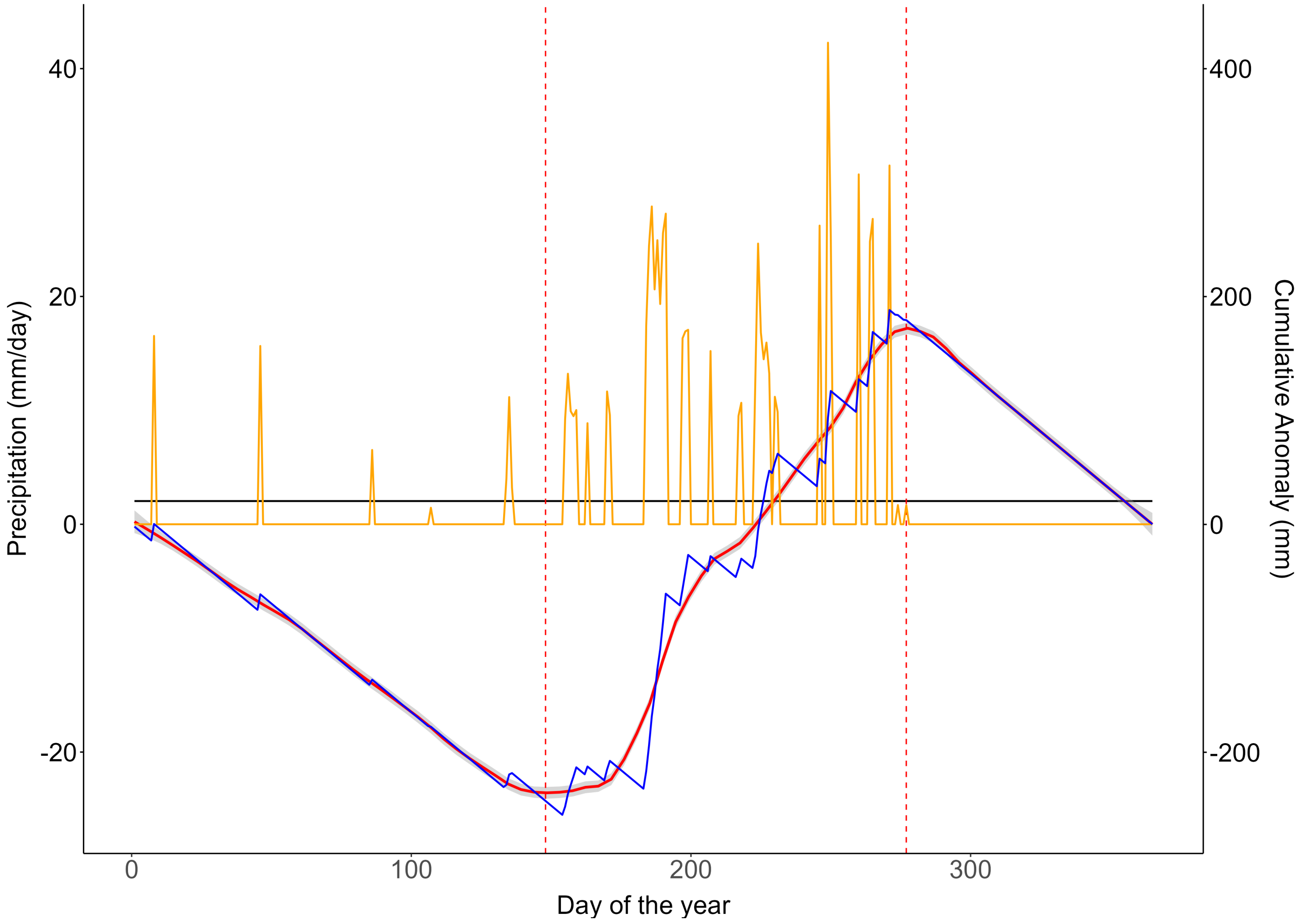


**Supplementary Figure S3.** LOWESS curve (solid red) fitted to cumulative precipitation anomaly data (blue) to estimate monsoon onset and demise. Original daily precipitation (orange) and annual mean daily rainfall (black) data shown for reference. Vertical dashed red lines represent the onset (day 148) and demise (day 277) estimates for an example climate grid cell in northern Bihar, 1995. Method adapted from Noska and Misra[13].

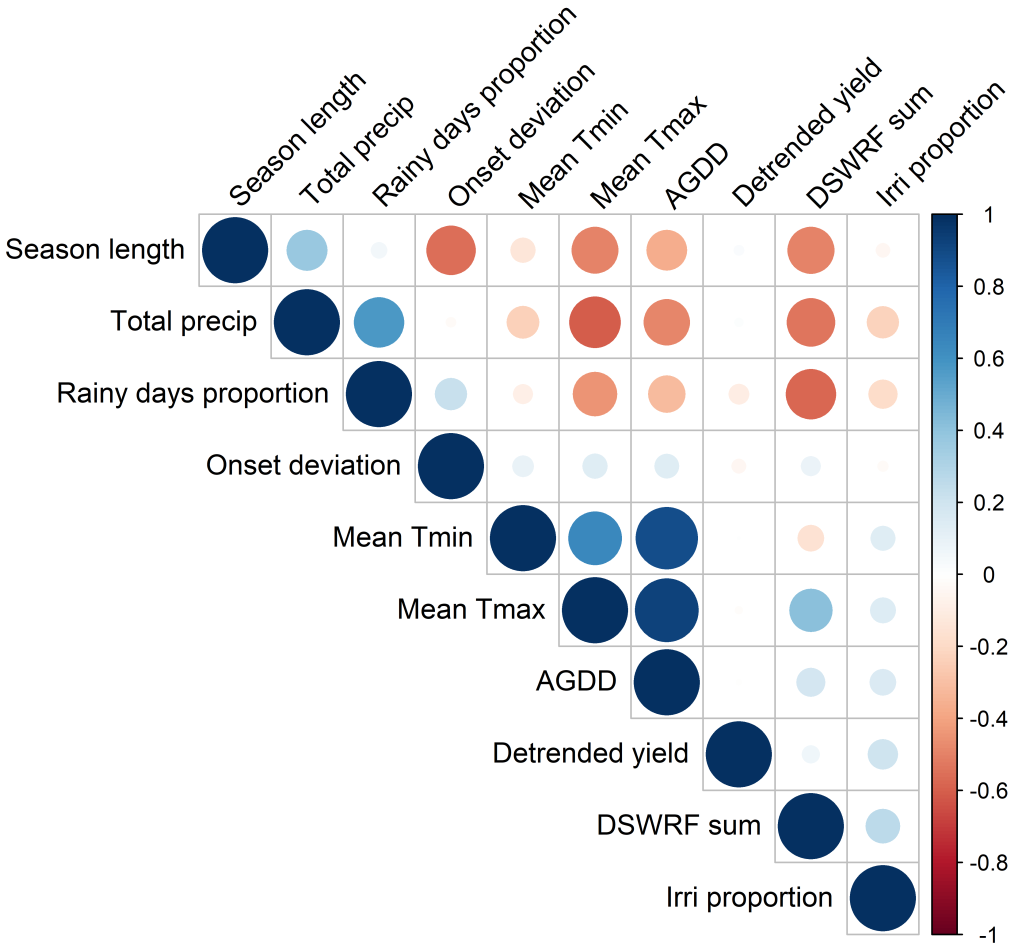


**Supplementary Figure S4.** Correlation matrix of all predictor and response variables.

## References


1. Frolking, S., Yeluripati, J. B. & Douglas, E. New district-level maps of rice cropping in India: A foundation for scientific input into policy assessment. *Field Crop. Res.* **98**, 164–177, DOI: 10.1016/j.fcr.2006.01.004 (2006).
2. Gumma, M. K., Nelson, A., Thenkabail, P. S. & Singh, A. N. Mapping rice areas of South Asia using MODIS multitemporal data. *J. Appl. Remote. Sens.* **5**, 053547, DOI: 10.1117/1.3619838 (2011).
3. Kar, G., Singh, R. & Verma, H. N. Alternative cropping strategies for assured and efficient crop production in upland rainfed rice areas of eastern India based on rainfall analysis. *Agric. Water Manag.* **67**, 47–62, DOI: 10.1016/j.agwat.2003.12.005 (2004).
4. Yadav, G. S. *et al.* Conservation tillage and nutrient management effects on productivity and soil carbon sequestration under double cropping of rice in north eastern region of India. *Ecol. Indic.* **105**, 303–315, DOI: 10.1016/j.ecolind.2017.08.071 (2019).
5. Webber, H. *et al.* No perfect storm for crop yield failure in Germany. *Environ. Res. Lett.* **15**, 104012, DOI: 10.1088/1748-9326/aba2a4 (2020).
6. Goulart, H. M. D., van der Wiel, K., Folberth, C., Balkovic, J. & van den Hurk, B. Storylines of weather-induced crop failure events under climate change. *Earth Syst. Dyn.* **12**, 1503–1527, DOI: 10.5194/esd-12-1503-2021 (2021).
7. Jeong, J. H. *et al.* Random Forests for Global and Regional Crop Yield Predictions. *PLOS ONE* **11**, e0156571, DOI: 10.1371/journal.pone.0156571 (2016).
8. Vogel, E. *et al.* The effects of climate extremes on global agricultural yields. *Environ. Res. Lett.* **14**, 054010, DOI: 10.1088/1748-9326/ab154b (2019).
9. Spera, S. A., Winter, J. M. & Partridge, T. F. Brazilian maize yields negatively affected by climate after land clearing. *Nat. Sustain.* **3**, 845–852, DOI: 10.1038/s41893-020-0560-3 (2020).
10. Lobell, D. B., Schlenker, W. & Costa-Roberts, J. Climate Trends and Global Crop Production Since 1980. *Science* **333**, 616–620, DOI: 10.1126/science.1204531 (2011).
11. Ray, D. K., Ramankutty, N., Mueller, N. D., West, P. C. & Foley, J. A. Recent patterns of crop yield growth and stagnation. *Nat. Commun.* **3**, 1293, DOI: 10.1038/ncomms2296 (2012).
12. Ray, D. K., Gerber, J. S., MacDonald, G. K. & West, P. C. Climate variation explains a third of global crop yield variability. *Nat. Commun.* **6**, 5989, DOI: 10.1038/ncomms6989 (2015).
13. Noska, R. & Misra, V. Characterizing the onset and demise of the Indian summer monsoon. *Geophys. Res. Lett.* **43**, 4547–4554, DOI: 10.1002/2016GL068409 (2016).
14. Moron, V. & Robertson, A. W. Interannual variability of Indian summer monsoon rainfall onset date at local scale. *Int. J. Climatol.* **34**, 1050–1061, DOI: 10.1002/joc.3745 (2014).
15. Boehm, R. *et al.* Association between Empirically Estimated Monsoon Dynamics and Other Weather Factors and Historical Tea Yields in China: Results from a Yield Response Model. *Climate* **4**, 20, DOI: 10.3390/cli4020020 (2016).
16. Kuhn, M. & Wickham, H. *Tidymodels: A Collection of Packages for Modeling and Machine Learning Using Tidyverse Principles.* (2020).
17. Barrios-Perez, C. *et al.* How does El Niño Southern Oscillation affect rice-producing environments in central Colombia? *Agric. For. Meteorol.* **306**, 108443, DOI: 10.1016/j.agrformet.2021.108443 (2021).
